# 金属有机框架材料用于水产品中危害物快速检测的研究进展

**DOI:** 10.3724/SP.J.1123.2026.02012

**Published:** 2026-11-08

**Authors:** Yiting ZHANG, Xiaohua XIAO, Gongke LI

**Affiliations:** 中山大学化学学院，广东 广州 510006; School of Chemistry，Sun Yat-sen University，Guangzhou 510006，China

**Keywords:** 金属有机框架材料, 水产品, 危害物, 快速检测, 样品前处理, metal-organic framework materials （MOFs）, aquatic products, contaminants, rapid detection, sample pretreatment

## Abstract

水产品含有丰富的优质蛋白质以及矿物质、脂肪酸和维生素等人体必需物质，具有丰富的营养价值，其生产和加工过程中易产生重金属、兽药残留、非法添加剂及致病微生物等危害物污染。快速检测技术是第一时间发现食品安全问题的重要手段。但水产品的基质复杂，现有快检技术缺少与之匹配的快速样品前处理技术和方法，容易导致“假阳性”或漏检等问题。金属有机框架材料（MOFs）凭借其超高的比表面积、大小可调节的孔径和易于功能化等独特优势，已成为水产品危害物分离分析领域中极具前景的新型材料。本文系统总结了2015‒2025年MOFs及其复合材料在水产品危害物快速检测中的应用研究进展。MOFs及其复合材料应用于固相萃取、固相微萃取、磁固相萃取、搅拌棒吸附萃取及QuEChERS等样品前处理技术，显著提升了水产品中危害物的选择性和富集效率，简化了前处理流程。MOFs与电化学、荧光光谱、表面增强拉曼光谱、免疫分析及适配体传感等多种检测技术结合，催生了一系列具有高灵敏度和高特异性的快速检测方法。此外，本文对MOFs在水产品分析领域面临的挑战和发展前景进行了总结与展望。

## Abstract (en)

Aquatic products are rich in high-quality protein and essential nutrients such as minerals，fatty acids，and vitamins，offering significant nutritional value. However，during their production and processing，they are susceptible to contamination by hazardous substances including heavy metals，veterinary drug residues，illegal additives，and pathogenic microorganisms. Thus，rapid detection technologies serve as a critical means for the timely identification of such food safety issues. However，the complex matrix of aquatic products presents significant analytical challenges，making separation and enrichment steps generally essential prior to detection. Current rapid detection methods often lack compatible rapid sample pretreatment approaches，which can lead to issues such as false positives or missed detection. Among the novel materials，metal-organic frameworks （MOFs） have emerged as a potential class of porous materials for the separation and detection of contaminations in aquatic products owing to their exceptional properties. Firstly，the ultra-high specific surface areas provide abundant active sites. Secondly，the uniform and tunable pore sizes can be tailored for molecular sieving and selective adsorption. Moreover，they are easy to functionalize through pre-design or post-synthetic modification，allowing the introduction of specific binding sites，catalytic moieties，or functional molecules. Lastly，MOFs are constructed through the coordination-driven self-assembly of metal ions and organic linkers，endowing them with considerable structural diversity that can be tailored for diverse applications. These attributes make MOFs exceptionally promising as advanced materials for tackling complex analytical challenges in food safety. This review provides a comprehensive and systematic summary of the application advances of MOFs and their composite materials in the rapid detection of contaminants in aquatic products over the past decade. In sample pretreatment，MOFs and their composites serve as superior adsorbents in various sample preparation techniques such as solid-phase extraction，solid-phase microextraction，magnetic solid-phase extraction，stir bar sorptive extraction，and QuEChERS. For solid-phase microextraction （SPME），MOFs coated on fibers enhance the extraction efficiency of volatile or semi-volatile compounds. In magnetic solid-phase extraction （MSPE），magnetic MOF composites enable rapid separation from liquid samples using an external magnet，significantly simplifying and expediting the process. These applications significantly enhance the selectivity and enrichment efficiency of contaminants in aquatic products while simplifying the pretreatment process. In rapid detection，MOF composites are used to develop enhanced detection platforms by leveraging their unique optical，electrochemical，and catalytic properties. In electrochemical analysis，conductive MOFs or MOF-modified electrodes amplify the signals of targets. For fluorescence detection，luminescent MOFs serve as sensitive probes，often in ratiometric modes to improve accuracy. MOFs also act as excellent substrates or enhancers in surface-enhanced Raman scattering （SERS） platforms，boosting Raman signals for ultra-sensitive detection of molecules. In immunoassays，MOFs are used as label carriers or signal amplifiers，improving detection limits for targets. Additionally，when integrated with aptamer-based biosensors，the large surface area of MOFs allows high-density immobilization of aptamers，enabling highly specific and sensitive detection of targets in complex matrices. These methods offer high sensitivity，specificity，and potential portability，providing innovative solutions for detecting trace contaminants in the complex matrix of aquatic products. Furthermore，this review discusses the challenges and future prospects of MOFs for the analysis of aquatic products. The significant challenges include the limited hydrolytic stability or chemical stability of certain MOFs in complex media，potential matrix effects in real samples that can affect performance，and the need for scalable and cost-effective synthesis of functionalized MOFs. Future efforts should focus on designing more stable and target-specific functionalized MOFs，promoting the integration of sample pretreatment and detection processes，and incorporating artificial intelligence for data analysis to establish an intelligent rapid detection system for aquatic product safety，which is of great significance for safeguarding public health.

水产品营养丰富^［1］^，可提供优质蛋白质及矿物质、脂肪酸和维生素等人体必需物质，发挥满足营养需求^［2］^和改善饮食结构的重要作用，深受人们喜爱。然而，水产品普遍存在的重金属^［3］^、致病微生物和生物毒素^［4］^、兽药残留^［5］^及非法添加剂等危害物正严重威胁食品安全与公众健康。其中，重金属污染主要来源于工业废水排放，兽药残留源于水产养殖过程中的违规用药，而非法添加剂主要存在于水产品加工流通环节，这些危害物可能引发胃肠道及神经系统症状，甚至危及生命。开发快速高效、灵敏准确的分析方法，实现水产品中危害物的精准检测，具有重要的现实意义。

我国已建立较完善的水产品危害物检测标准。GB 31650-2019规定，在水产动物及其制品中地西泮不得检出；GB 31650.1-2022规定，氧氟沙星残留限量为2 μg/kg；GB 2762-2022规定，水产品中多氯联苯的残留量不得多于20 μg/kg。高效液相色谱-质谱联用（HPLC-MS）和气相色谱-质谱联用（GC-MS）是水产品危害物检测的金标准，但复杂耗时的样品前处理以及对大型仪器的依赖，限制了其应用范围，尤其是在现场快速筛查中的应用^［6］^。发展高效、简便且具有高选择性的样品前处理技术，如固相萃取（SPE）、固相微萃取（SPME）和磁固相萃取（MSPE）等，是实现现场快速准确检测的重要前提；另一方面，以电化学方法、光谱法、免疫学方法和适配体传感方法为代表的快速检测技术，具有响应速度快、灵敏度高和便携化程度高的优势^［7］^。

开发新型功能材料对于提升检测方法的性能至关重要。具有极低室温蒸气压和良好热稳定性的离子液体^［8］^、具有高度特异性的分子印迹聚合物（MIPs）^［9］^、结构和特性丰富的碳纳米材料^［10］^，以及金属有机框架（MOFs）材料^［11-13］^等均得到了广泛研究。在水产品安全分析领域，已有综述系统总结了不同样品前处理技术及多种先进材料的应用^［14，15］^和基于纳米材料的多种检测技术^［7］^，并重点关注了贵金属纳米颗粒与MOFs等纳米材料。MOFs是一类由金属中心和有机配体桥联而成^［16］^的晶态多孔化合物，其核心优势在于极高的孔隙率与大比表面积，为吸附富集目标物提供了充足的结合位点；得益于可精细调节的孔径，MOFs可实现对特定尺寸危害物的选择性识别；开放的金属位点^［17］^还使其易于修饰，可根据目标分析物的结构特征，通过优化金属中心、有机配体种类或合成策略进行定制化改性^［18］^，赋予材料特异性识别能力，提升抗干扰性能，在水产品危害物检测中应用广泛（图1）。尽管MOFs种类丰富，但受限于稳定性，仅有部分能被用于分离和检测^［19］^。MIL-n系列由过渡金属和羧酸配体桥联而成，具有高比表面积和孔隙率；UiO-n系列以锆为核心，凭借极高的化学稳定性、简便的合成工艺及低成本特性成为研究热点；ZIF-n系列则通过模拟沸石拓扑结构，利用锌或钴与咪唑配体连接，兼具出色的吸附性能与结构稳定性；而PCN-n系列以其多样的结构与强功能可调性在复杂分析场景中表现突出；吉林大学的JUC-n系列作为功能导向材料，同样具有多样的结构。MOFs的合成通常采用溶剂热法，通过改变反应条件可实现对其晶体尺寸和形貌的调控，其中溶剂和温度是关键因素；微波辅助或超声辅助可以加速合成反应^［20］^。但是，多数MOFs存在水稳定性和选择性不佳以及可回收性差等不足。通过原位引入功能位点，或采用共价后修饰和配位后修饰等后合成修饰法在母体框架上嫁接功能基团^［21］^，MOFs的性能可得到进一步优化。提升MOFs的稳定性与可回收性已成为该领域的重要研究方向^［22］^。

**图1:**
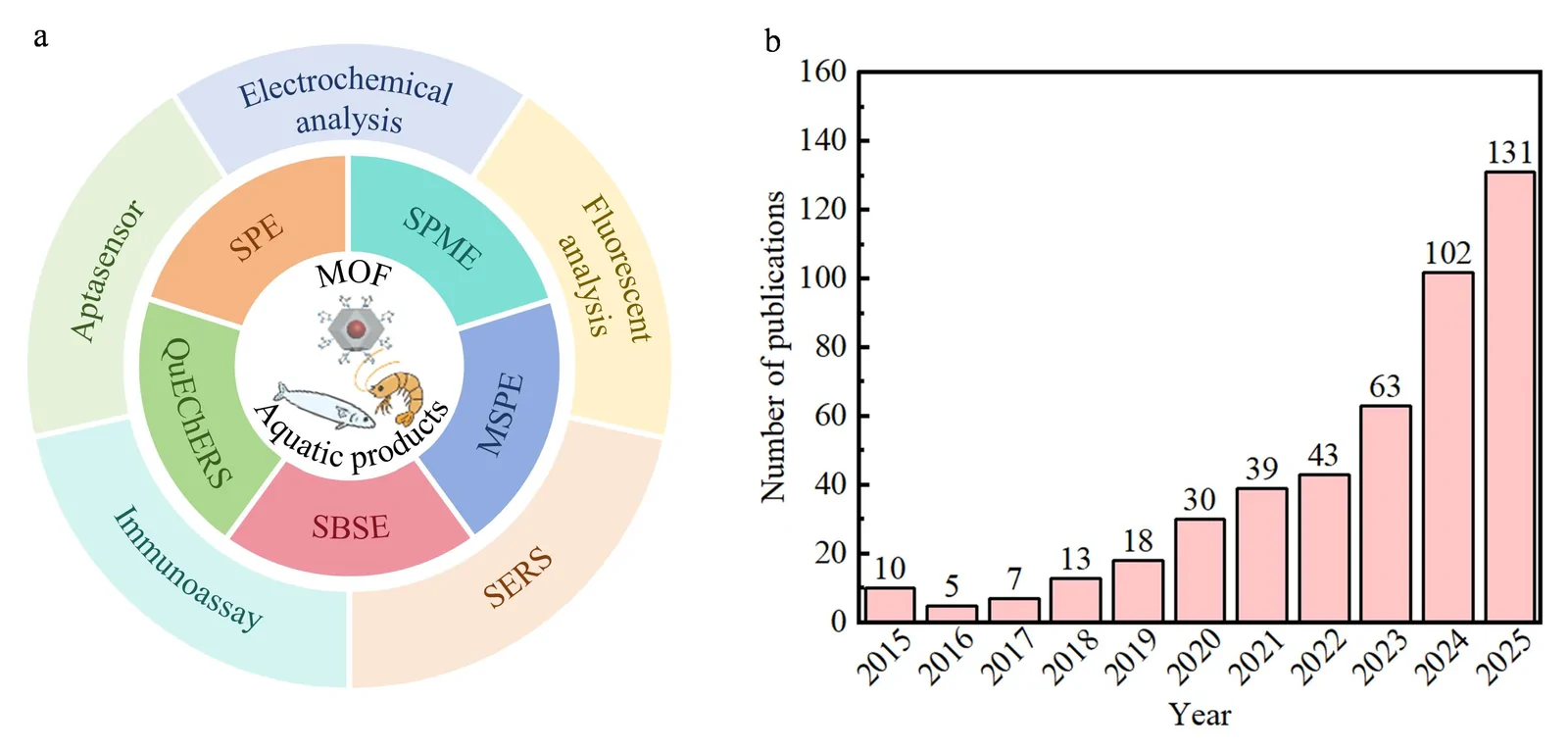
（a）金属有机框架材料在水产品危害物分离分析中的应用；（b）近十年MOFs材料应用于水产品危害物分离分析领域中的文献数量变化趋势 Data source： Web of Science Core Collection； search year： 2015‒2025； keywords： metal organic framework，MOFs，aquatic product，seafood，fish，shellfish，shrimp，aquaculture. SBSE： stir bar sorptive extraction； SERS： surface-enhanced Raman spectroscopy.

已有研究综述了基于MOFs的电化学传感^［23］^和荧光检测技术^［24］^在水产品检测中的应用。为应对水产品快速检测的实际需求，本文系统梳理了MOFs在水产品的样品前处理领域及多种前沿检测技术中的研究成果。

## 1 基于金属有机框架材料的样品前处理技术

水产品基质复杂，含有较多内源性干扰物质^［25］^，因此在分析前对样品进行前处理以纯化、富集目标物^［26］^至关重要，该步骤直接影响检测的选择性与灵敏度。MOFs的高比表面积提供高效的富集能力，可缩短萃取时间；其孔道结构的尺寸筛分效应可实现特定尺寸危害物的选择性捕获，排除基质中大分子干扰物，从而简化前处理步骤。相较传统的C18吸附剂，MOFs的萃取时间缩短了一半以上。然而，MOFs选择性不足及水稳定性较差的缺陷，限制了其在水产品复杂基质中的直接应用。ZIF系列以及以UiO系列和PCN系列为代表的Zr-MOFs因其优异的化学稳定性和水稳定性，在生物基质的样品前处理中表现优异。通过对MOFs进行功能化改性，可显著提升材料的选择性与稳定性，拓展其在实际检测中的应用。

### 1.1 固相萃取技术

MOFs作为固相萃取的新型吸附剂，可通过独特的分子尺寸排阻效应^［27］^同步实现目标物的富集与基质干扰的排除。此外，其有机配体中的芳香环可与芳香族目标物形成*π-π*堆积作用，配体中常含有的氨基、羧基和羟基等官能团还可与分析物中的极性基团形成氢键。部分MOFs含有配位不饱和的金属开放位点，可作为路易斯酸与富电子目标物发生配位作用。上述机制的协同作用共同决定MOFs对特定目标物的萃取效率与选择性。

PCN-222通过其卟啉配体形成的配位键，可增强对Hg（Ⅱ）的选择性吸附，且材料本身可在较宽的pH范围内保持结构完整，当其用于鱼样中的汞离子萃取时，富集因子可达120倍^［28］^。UiO-66具有高化学稳定性和热稳定性，其多孔框架结构有利于目标物的扩散和吸附。利用单宁酸对UiO-66-NH_2_进行改性，单宁酸中的酚羟基与Pb（Ⅱ）离子产生强配位作用，使得该吸附剂对Pb（Ⅱ）的理论吸附容量达到475.78 mg/g；将其用于水产品中Pb（Ⅱ）的富集，结合激光诱导击穿光谱（LIBS）检测，可使Pb（Ⅱ）的检出限低至0.18 μg/mL^［29］^，低于国标规定的0.5 μg/mL。

氨基化的UiO-66与弱酸性非甾体抗炎药间存在较强的静电相互作用和*π-π*相互作用，可用于鱼虾肌肉组织中5种常见非甾体抗炎药的高效富集^［30］^。而全氟化的UiO-66则通过与全氟和多氟烷基物质（PFAS）间的氟-氟相互作用，显著提升了材料对虾样品中PFASs的选择性，尤其是短链PFASs^［31］^。

综上，基于MOFs材料的固相萃取技术在水产品危害物分析中的应用归纳如表1所示。

**表1:** 基于MOFs材料的固相萃取技术在水产品危害物分析中的应用

Main material	Analytical method	Analytes （samples）	Extraction time/min	LOD/（ng/mL）	Recovery/%	Ref.
MOF-5@CS	UHPLC-MS/MS	biotoxin （fish）	30	0.0018‒0.077	84.36‒118.51	［^32^］
MIL-101@MIP	HPLC	CAP （clam，fish，shrimp）	22.5	0.08	75.0‒103.7	［^33^］
Fe_3_O_4_@DMcT/MIL-101（Fe）	CVAAS	Hg（Ⅱ） （fish，shrimp，tuna）	14	0.01	83.3‒106	［^34^］
UiO-66-F_4_/PAN	UHPLC-HRMS	PFAS （shrimp）	12	0.008‒0.076	60.98‒122.83	［^31^］
CF@UiO-66-NH_2_	UHPLC	NSAID （shrimp，fish）	33	0.12‒3.50	72.95‒116.99	［^30^］
UiO-66-NH_2_@tannic acid	LIBS	Pb（Ⅱ） （croaker，scallop）	30	180	75.25‒99.08	［^29^］
PCN-222	CVAAS	Hg（Ⅱ） （fish）	7	0.02	74.3‒98.7	［^28^］
DUT-67	CE-DAD	organic mercury （fish）	\	0.022‒0.067	83.2‒92.4	［^35^］
Tb-MOF	SP	MG （fish）	120	1.66	95.6‒99.3	［^36^］

CS： chitosan； MIP： molecular imprinting polymer； DMcT： 2，5-dimercapto-1，3，4-thiadiazole； PAN： polyacrylonitrile； CF： cotton fibers； CVAAS： cold vapor atomic absorption spectroscopy； LIBS： laser-induced breakdown spectroscopy； CE： capillary electrophoresis； DAD： diode-array detection； SP： spectrophotometry； CAP： chloramphenicol； PFAS： per- and polyfluoroalkyl substance； NSAID： nonsteroidal antiinflammatory drug； MG： malachite green.

### 1.2 固相微萃取技术

在SPE的基础上发展而来的固相微萃取技术无需溶剂，且对样品量需求较少^［37］^，具有更低的检出限和检测成本^［38］^。SPME涂层的制备方法多样，主要包括物理涂渍法、原位生长法和溶胶-凝胶法。其中，物理涂渍法操作便捷，但涂层稳定性较差；原位生长法则通过化学键合和电化学法等方式使涂层材料在基底表面直接生长，展现出更优的萃取性能和稳定性；溶胶-凝胶法可制备多孔且高比表面积的涂层，稳定性好，但制备过程耗时较长，且易产生裂纹。将MOFs涂层通过上述方法固定于纤维基底上，可克服聚二甲基硅氧烷和聚丙烯酸酯等商业涂层性能有限的问题。涂层均匀致密、厚度可控且结合牢固，确保了萃取效率的稳定。

虽然UiO-66具有高比表面积和多孔结构，但1~2 nm的孔径范围使其无法有效捕获多环芳烃（PAH）。将UiO-66与层状的二硫化钼（MoS_2_）结合，形成分级多孔结构，能够高效捕获多种尺寸的16种PAHs。UiO-66/MoS_2_的SPME复合涂层用于鱼样中PAHs的顶空萃取，可有效规避MOFs的水稳定性缺陷，其吸附容量达到了商业涂层的2.1~4.5倍^［39］^。

ZIF-8具有较大的内孔径，以及良好的疏水性、热稳定性和耐碱性。将衍生化试剂封装在ZIF-8的孔隙中，可保护衍生化试剂不被水解，实现SPME纤维上的衍生化，40 ℃下即可完成非挥发性脂肪族二胺的水相萃取^［40］^。ZIF-8的*π-π*堆积作用增强了对雌激素的吸附能力，将分子印迹聚合物与磁性ZIF-8结合，可同时解决MOFs选择性不足的缺陷以及MIPs材料在吸附容量和传质效率上的局限^［41］^，对鱼样中雌激素的吸附容量可提高2.3~3.4倍，实现24个样品并行的自动化SPME^［42］^。

综上，基于MOFs材料的固相微萃取技术在水产品危害物分析中的应用归纳如表2所示。

**表2:** 基于MOFs材料的固相微萃取技术在水产品危害物分析中的应用

Main material	Analytical method	Analyte （samples）	Extraction time/min	LOD/（ng/mL）	Recovery/%	Ref.
NH_2_-MIL-53（Al）	UHPLC-MS/MS	sulfonamide （fish）	\	0.0013‒0.0047	85.6‒113	［^43^］
MIL-101（Cr）-NH_2_	LC-MS/MS	antibiotic （fish）	10	0.2‒1.1	\	［^44^］
UiO-66/MoS_2_	GC-MS/MS	PAH （fish）	30	0.00011‒0.0014	80.2‒101	［^39^］
ZIF@PPHF	HPLC	histamine （fish）	7	19	97‒102.8	［^45^］
ZIF-8	GC-MS	BA （fish）	40	27.1‒33.2	78.6‒104	［^40^］
Fe_3_O_4_@ZIF-8@MIP	HPLC	estrogen （fish）	25	0.4‒1.7	74.5‒95.3	［^42^］
aptamer@ZIF-8	HPLC-UV	DA （clam，mussel）	10	7.0	91.2‒94.5	［^46^］
Cu-DAT-PI	GC	PAH （fish）	60	1.3‒6.7	\	［^47^］
*β*-CD-Cu-MOF/cellulose	GC-MS	BA （fish）	6	0.03‒0.11	95.0‒99.6	［^48^］

SPME： solid-phase microextraction； MoS_2_： molybdenum disulfide； PPHF： polypropylene hollow fiber； DAT： 2，5-diaminoterephthalate； PI： polyimide； *β*-CD： *β*-cyclodextrin； PAH： polycyclic aromatic hydrocarbon； BA： biogenic amine； DA： domoic acid.

### 1.3 磁固相萃取技术

传统SPE依赖于萃取柱，包含活化、上样、淋洗、洗脱等多个步骤，耗时长且可能发生柱堵塞。而MSPE将磁性吸附剂直接分散于样品溶液中，使吸附剂与目标物充分接触，再利用外部磁场完成样品分离，简化了样品预处理过程；且磁性吸附剂在溶液中高度分散，传质阻力小，可在更短时间内达到吸附平衡。此外，传统SPE的自动化仍受限于有限的柱容量，而MSPE自动化可实现对多批次样品的高通量净化。磁性MOFs复合材料继承了MOFs的高比表面积和丰富孔道，可提供高吸附容量；其纳米颗粒在溶液中具有良好的分散性，确保了与目标物的充分接触。此外，MOF壳层可通过功能化修饰增强对特定目标物的亲和力。

未经修饰的ZIF-8因表面的锌位点被有机配体完全配位，导致吸附容量较低。利用锌离子溶液进行后合成修饰，可在材料表面创造Zn^2+^活性位点，从而与软骨藻酸（DA）中的氧原子和氮原子形成配位键。磁性ZIF-8/Zn^2+^微球能实现5 min内DA分子的快速吸附（图2），结合LC-MS/MS在贝类样品中检出了49.2 pg/mL的软骨藻酸^［49］^。针对同一问题，通过多层结构设计制备了Fe_3_O_4_@（ZIF-8）_3_，其吸附容量相较单层的Fe_3_O_4_@ZIF-8提高了39.2%，结合单微球免疫传感器用于鱼样中氯霉素的高灵敏检测时，检出限低至46.42 pmol/L^［50］^，远低于国标检出限310 pmol/L。

**图2:**
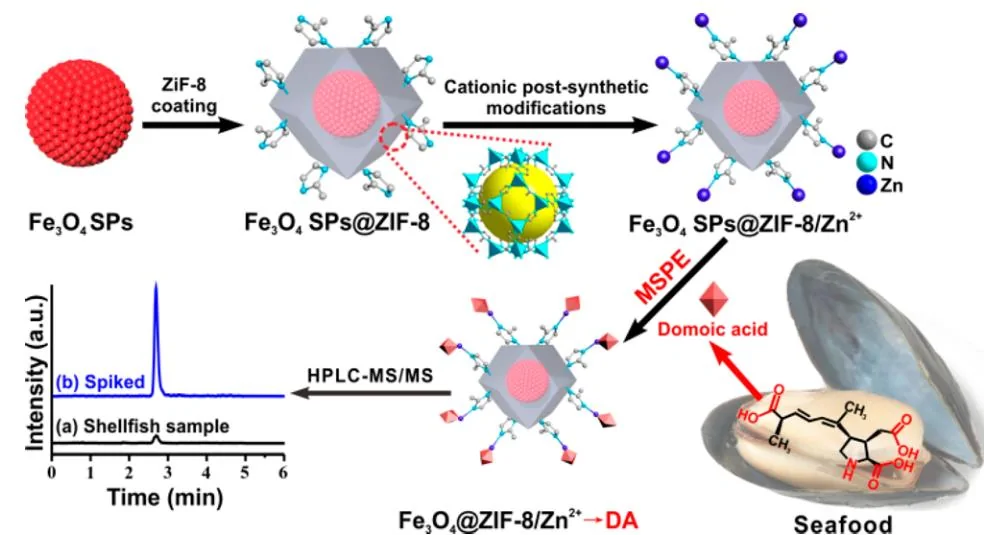
Fe_3_O_4_ SPs@ZIF-8/Zn^2+^的制备以及贝类中软骨藻酸的MSPE-HPLC-MS/MS过程^［49］^

UiO-66具有大量的Zr^4+^金属节点，与软骨藻酸分子上带负电的羧酸基团之间存在强静电相互作用。将磁性核壳微球Fe_3_O_4_@SiO_2_@UiO-66用于DA萃取，结合HPLC-MS/MS在贝类样品中实现1.45 ng/L的低检出限，且RSD小于5.0%^［51］^。与大多数具有孔径限制的微孔MOFs不同，JUC-48内部的一维六边形纳米管道赋予其介孔特性，有利于萃取尺寸较大的分子。磁性JUC-48材料已被用于萃取虾肉样品中的5种磺胺类药物^［52］^。

综上，基于MOFs材料的磁固相萃取技术在水产品危害物分析中的应用归纳如表3所示。

**表3:** 基于MOFs材料的磁固相萃取技术在水产品危害物分析中的应用

Main material	Analytical method	Analytes （samples）	Extraction time/min	LOD/（ng/mL）	Recovery/%	Ref.
Fe_3_O_4_@PEI-MOF-5	UHPLC-MS/MS	MG，CV （fish）	40	0.30，0.08	83.15‒96.53	［^53^］
IRMOF-3@SiO_2_/Fe_3_O_4_	LC-MS/MS	quinolone （fish）	10	0.02‒0.07	80.4‒112	［^54^］
Fe_3_O_4_@SiO_2_@MIL-53（Al）-NH_2_	HPLC-DAD	estrogen （fish）	5	0.091‒0.52	79.3‒115.2	［^55^］
Fe_3_O_4_@SiO_2_@UiO-66	HPLC-MS/MS	DA （shellfish）	6	0.00145	93.1‒107.3	［^51^］
Fe_3_O_4_ SPs@ZIF-8/Zn^2+^	LC-MS/MS	DA （shellfish，fish，crayfish，crab）	5	0.0002	93.1‒102.3	［^49^］
Fe_3_O_4_@ZIF-8&F_13_	LC-MS/MS	PFCA （fish）	5	0.005‒0.021	88.4‒107.1	［^56^］
Fe_3_O_4_@（ZIF-8）_3_	immunosensor	CAP （fish）	20	0.015	82.00‒113.29	［^50^］
Fe_3_O_4_@JUC-48	HPLC-DAD	sulfonamide （shrimp）	8	1.73‒5.23	79.2‒102.5	［^52^］
Fe_3_O_4_-pyridine@Cu_3_（BTC）_2_	FAAS	Cd（Ⅱ），Pb（Ⅱ） （fish）	14	0.2，1.1	92.8‒97	［^57^］

MSPE： magnetic solid phase extraction； PEI： polyethyleneimine； BTC： 1，3，5-benzenetricarboxylic acid； FAAS： flame atomic absorption spectrometry； CV： crystal violet； PFCA： perfluoroalkyl carboxylic acid.

### 1.4 搅拌棒吸附萃取技术

搅拌棒吸附萃取（SBSE）将搅拌棒涂层作为萃取相，弥补了SPME纤维易碎及容量有限^［58］^的不足。萃取涂层是决定SBSE吸附性能的关键，传统的PDMS涂层对极性化合物吸附效率低^［59］^，功能化设计的MOFs显著拓宽了SBSE的适用范围。

MOF-5（Fe）中的Fe^3+^与多氯联苯（PCBs）中的氯原子间存在静电相互作用。将磁性MOF-5（Fe）复合材料作为SBSE涂层用于鱼样中PCBs的萃取，相较PDMS等商业涂层，选择性显著提升^［60］^。使用适配体对MOF-5进行功能化，能增强材料对PCBs的特异性识别能力^［61］^。

喹诺酮类在酸性条件下主要以阳离子形式存在。利用PCN-224配体中羧基的电荷相互作用，将PCN-224涂覆棉线包裹的悬浮搅拌棒用于鱼类组织中喹诺酮药物的萃取，在有效降低基质干扰的同时，提高了检测的灵敏度（图3）^［62］^。UiO-66-（OH）_2_表面具有呈负电性的羟基，能与喹诺酮药物产生静电引力和分子间氢键作用；羟基功能化还增强了材料的水稳定性，使其适用于复杂生物样品，从而提高了鱼和虾样品中痕量喹诺酮类抗生素的吸附效率^［63］^。

**图3:**
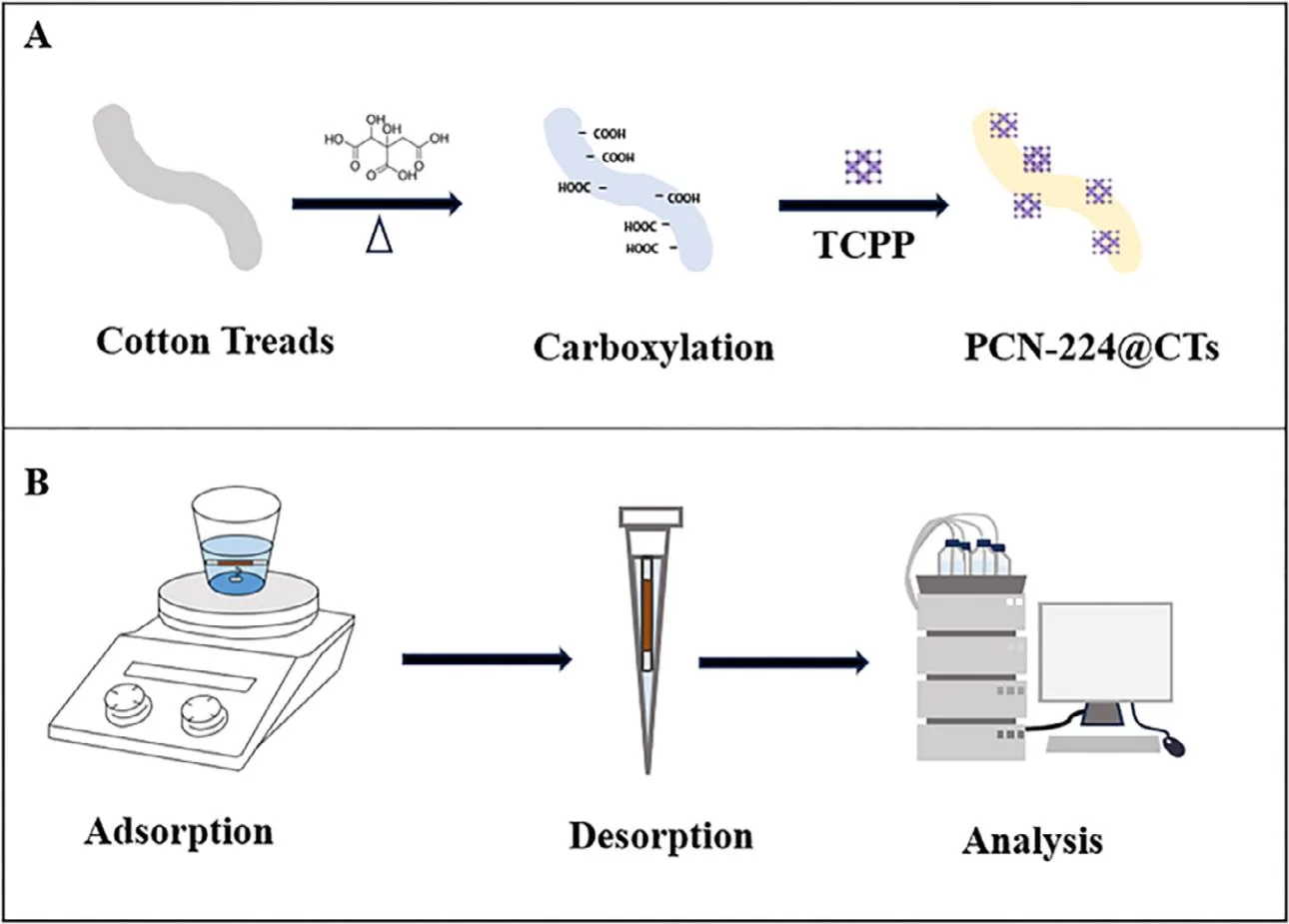
PCN-224@CTs的制备及搅拌棒吸附萃取过程^［62］^ CTs： cotton threads.

### 1.5 QuEChERS技术

QuEChERS技术简便、高效，适合多残留分析，但传统吸附剂在复杂基质背景和多组分高通量分析中性能有限。MOFs材料的应用可以提升材料分散性，有效去除干扰，提高复杂基质的纯化效率^［64］^。

ZIF-67结构中的咪唑环对三苯甲烷类染料有天然的亲和力，而聚丙烯酸提供带负电的羧基，能通过静电相互作用吸附阳离子染料。将两种材料复合，并结合具有亲水性和结构柔性的聚醚砜，可形成新型的多孔QuEChERS吸附剂，显著提高对鱼样中孔雀石绿和结晶紫的吸附选择性，吸附率分别达99.11%和99.03%^［65］^。

## 2 基于金属有机框架材料的快速检测技术

用于水产品中危害物分析的快速检测技术种类丰富。化学技术，包括电化学技术和光谱技术，主要利用物理和化学性质的差异实现对目标分析物的检测。生物检测技术则基于特异性的生物分子识别，常结合高灵敏度的化学信号放大技术。此外，为简化检测流程、提升检测的准确度和灵敏度，快速检测技术正朝着前处理与检测一体化的方向发展。

MOFs兼具高效的富集能力、灵活的功能化特性及多样的信号传导机制，成为水产品快速检测的理想材料选择。这些特性能够有效解决水产品基质复杂的问题，实现对痕量目标物的快速检测，展现出巨大的应用潜力。例如，MOFs具有极强的吸附能力且易于功能化，能够通过强相互作用与化学功能分子和生物识别元件相结合；部分MOFs本身就具有荧光特性，且与传统染料相比毒性更低；此外，MOFs还可作为SERS方法中的拉曼增强基底^［66］^。

### 2.1 电化学分析技术

电化学分析以电极作为检测元件，利用不同物质间电化学性质的差异，根据电信号变化进行检测，需要电子传导能力优异且电催化活性良好的电极修饰材料。MOFs材料常用于修饰电极或作为材料载体，也可作为前驱体原位生成其他纳米材料，其多孔性可以改善电化学传感的过程，同时实现分析物的吸附。为改善MOFs电导率不佳^［67］^的限制，主要可采用3种方式。其一是合成本征导电MOFs，通过选用含硫、氮等原子的共轭有机配体，或在骨架中引入混合价态金属离子，提升电荷传输能力；其二是将MOFs与金属纳米粒子、碳材料或导电聚合物等具有良好导电性的材料复合，通过客体的电荷传输改善导电性；最后，还可将MOFs作为前驱体在惰性气氛下高温热解，获得保留多孔结构的金属/碳复合材料。

MOFs材料中金属离子与有机配体间的配位键在高温条件下不稳定，利用这一特点，MOFs可以作为金属氧化物/碳复合材料的合成前驱体^［68］^。经Ni-MOF热解碳化制备的Ni@C材料在保留叶片状结构的同时使多孔碳均匀分布，增大了有效表面积和活性位点数量，为组胺吸附和电子转移提供了充足空间，成功用于鱼样中组胺的电化学传感检测^［69］^。

Cu-MOF具有丰富的表面官能团，活性位点多。Cu-MOF结构中的亚胺基团及羟基可有效富集Hg^2+^至玻碳电极（GCE）表面；且其合成过程中形成的Cu_2_O纳米颗粒显著增强了材料导电性，使得GCE/Cu-MOF电化学传感平台成功用于罐装金枪鱼中Hg^2+^的检测^［70］^。其检出限为0.063 3 nmol/L，远低于国标方法的检出限50 nmol/L。硫脲分子中的氨基与培氟沙星间存在氢键作用。使用硫脲修饰Cu-MOF，结合具有良好导电性的多壁碳纳米管（MWCNT），能够设计出用于虾肉中培氟沙星检测的便携式电化学传感器^［71］^。

由于协同效应，双金属MOFs相比单金属MOFs具有更高的电导率和更丰富的氧化还原特性。Mn^2+^和Co^2+^具有相似的离子半径和电负性，使得Mn-Co-MOF结构均匀，且其多级孔结构有利于组胺分子的扩散。基于Mn-Co-MOF与碳纳米纤维复合材料构建的电化学传感器，可有效检测不同储存时间下鱼样中组胺含量^［72］^。利用MXene的高导电性与CeFe-MOF的比表面积优势，引入氨基基团增强对重金属离子的特异性吸附，能够制备高灵敏电化学传感器，同时检测鱼样中的不同重金属离子^［73］^。

综上，基于MOFs材料的电化学分析技术在水产品危害物分析中的应用归纳如表4所示。

**表4:** 基于MOFs材料的电化学分析技术在水产品危害物分析中的应用

Main material	Analytes （sample）	Analytical time/min	LOD/（nmol/L）	Recovery/%	RSD/%	Ref.
Ni@C/GCE	histamine （fish）	\	0.32	96.9‒104.6	2.9	［^69^］
Ca-MOF	Hg（Ⅱ） （fish oil）	6	2.99	98±5	3.8	［^74^］
Nb（BTC）MOF@CNF	purine derivative （fish）	\	68‒76	99.3‒101.1	1.1‒1.8	［^75^］
MIP-Au@Fe-BDC/N，S-GQDs/GCE	histamine （tuna）	21	0.026	97.2‒102.1	2.5‒3.8	［^76^］
GCE/Cu-MOF	Hg（Ⅱ） （tuna）	5‒10	0.0633	115.5‒131.8	\	［^70^］
Au-AgMSs@Cu-MOF	bisphenol （tuna）	5	2.9‒3.2	94	2.15	［^77^］
Cu-BTC@FeNi-SAs/MIP	sulfadiazine （fish）	15.5	0.00114	91.02‒98.10	0.54‒1.32	［^78^］
Ag/Cu-MOF	MG （fish）	1.5	2.2	\	3.7	［^79^］
Th-Cu-MOF/MWCNTs	pefloxacin （shrimp）	4	2.6	95.87‒104.4	1.1‒3.1	［^71^］
Mn-Co（2-MeIm）MOF@CNF	histamine （fish）	1‒5	89.6	99.7‒100.3	0.89‒1.68	［^72^］
MXene-NH_2_@CeFe-MOF-NH_2_	Cd，Pb，Hg （fish）	6‒7	0.33‒0.95	97‒110.85	0.93‒3.34	［^73^］
Cu-HHTP@Zn-TCPP	H_2_S （shrimp）	<1	620	99.57‒100.81	<3	［^80^］

GCE： glassy carbon electrode； CNF： carbon nanofibers； BDC： benzene-1，4-dicarboxylate； GQDs： graphene quantum dots； MSs： microstructures； SAs： single atoms； MWCNTs： multi-walled carbon nanotubes； MeIm： 2-methylimidazole； HHTP： hexahydroxytriphenylene； TCPP： tetra（4-carboxyphenyl）porphyrin.

### 2.2 荧光分析技术

荧光分析根据检测物质的荧光信号变化进行检测，需要强且稳定、同时对目标物响应灵敏的荧光发射。具有高比表面积和高负载能力的MOFs可以同时用作靶标的吸附剂和荧光信号的载体。例如以镧系金属为中心的MOFs斯托克斯位移大、荧光寿命长、荧光发射无干扰且发射带窄^［81］^，被认为是荧光传感的理想材料^［82］^。

MOFs用于荧光检测时，根据荧光信号来源通常可分为3类。其一即使用具有本征荧光的MOFs材料，可直接使用发光金属离子作为配位中心，也可以利用含荧光发色团（如芳香骨架或*π*共轭结构）的有机配体^［83］^。Cu-MOF的强荧光发射源于金属到配体的电荷转移过程，与挥发性生物胺作用后发生荧光猝灭，其荧光强度随生物胺浓度增加而降低。基于Cu-MOF的传感纸盘能够实现不同温度条件下鲈鱼样品中挥发性生物胺的实时监测，检出限低且选择性好^［84］^。四环素盐酸盐（TCH）能有效猝灭Eu-BDC的红色荧光。通过在Eu-BDC中掺杂具有稳定绿光发射的Tb^3+^，可建立比率荧光信号变化，实现鲫鱼和蛤仔样品中TCH的高灵敏度检测^［85］^。

构建荧光MOFs的第2种方法是将发光的客体分子封装于MOFs材料中^［86］^，常见的发光材料有量子点（QDs）和荧光染料等。ZIF-8中的Zn^2+^对氯四环素药物具有荧光敏化效应，使氯四环素的绿色荧光增强；而氯四环素对CdTe QDs的内滤效应又导致该量子点的红色荧光减弱。基于此原理构建的CdTe QDs@ZIF-8比率型荧光传感器对巴沙鱼中氯四环素的响应时间仅1 min，且表现出优异的可重复使用性^［87］^。Fe-CDs@MIL-101（Fe）复合纳米酶可结合邻苯二胺与黄嘌呤氧化酶构建比率荧光探针，用于检测鱼样中的黄嘌呤^［88］^。鱼类腐败产生的黄嘌呤被其氧化酶氧化，产生H_2_O_2_；MIL-101（Fe）具有类过氧化酶活性，可催化邻苯二胺和H_2_O_2_反应，生成的物质能够猝灭Fe掺杂碳点（Fe-CDs）的蓝色荧光。该探针还能够识别鱼类冷冻储存过程中的早期腐败迹象。

利用MOFs的孔限域和吸附作用，可诱导物质产生聚集诱导发光（AIE）效应。例如，Zn-BTEC的多孔结构可促使氯四环素（CTC）分子扩散进入并发生聚集，从而引发荧光增强；且该材料只有在结合氯四环素时可产生独特的荧光发射波长，有效区别于其他四环素类抗生素。因此，Zn-BTEC可应用于鱼样品中氯四环素的选择性测定（图4）^［89］^。由于双层MOFs的荧光猝灭效率优于单层MOFs，Yang等^［90］^构建了一种基于MOF-on-MOF异质结构材料（Zn-BTEC@ZIF-8@TCH）的免标记荧光传感器，用于检测鱼样中的组胺。ZIF-8的微介孔特性可充分吸附TCH分子，引发AIE效应；而组胺与MOF配位会导致该结构坍塌，引发荧光猝灭。

**图4:**
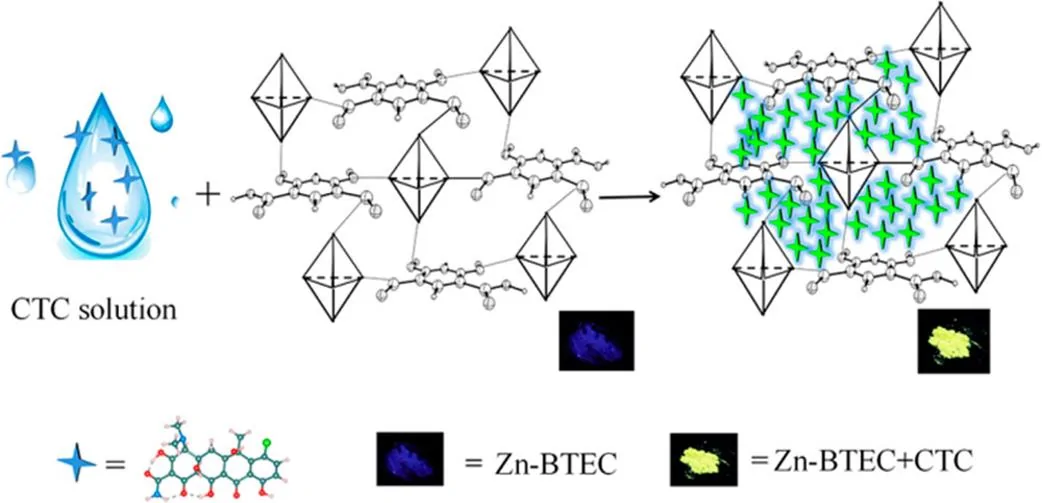
基于AIE机理，利用金属有机框架Zn-BTEC作为荧光探针对氯四环素进行传感过程的示意图^［89］^ AIE： aggregation-induced emission； CTC： chlorotetracycline.

综上，基于MOFs材料的荧光分析技术在水产品危害物分析中的应用归纳如表5所示。

**表5:** 基于MOFs材料的荧光分析技术在水产品危害物分析中的应用

Main material	Analyte （samples）	Analytical time/min	LOD/（μmol/L）	Recovery/%	RSD/%	Ref.
Fe-CDs@MIL-101（Fe）	xanthine （fish）	30	0.36	95.5‒109	1.7‒9.3	［^88^］
Ru@UiO-OH	BA （shrimp，fish）	1‒5	0.000389	\	\	［^91^］
CdTe QDs@ZIF-8	chlortetracycline （fish）	1	0.037	95.0‒106.0	0.5‒2.4	［^87^］
GSH-AuNCs/ZIF-8	Cu（Ⅱ） （fish）	\	0.08	89.2‒103.0	1.7‒3.9	［^92^］
GQDs/AuNCs@ZIF-8	histamine （fish，shrimp，shellfish）	2.3	7.49	\	\	［^93^］
Zn-BTEC@ZIF-8@TCH	histamine （fish）	20	13.12	103.3‒115.0	2.4‒4.5	［^90^］
Zn-BTEC	chlortetracycline （fish，clam）	\	0.028	107.2‒108.5	1.4‒4.3	［^89^］
Al-MOF，RhB	MG （fish）	3‒5	4.86	81.90‒108.00	1.00‒4.69	［^94^］
Tb-MOF	MG （fish，shrimp）	1‒2	0.0108	99.8‒107.99	1.1‒2.2	［^95^］
Ce-MOF	H_2_S （shrimp，salmon）	2	0.156	\	\	［^96^］
Sm-DDB	inorganic phosphori （shrimp，fish）	2	0.38‒1.33	93.02‒104.11	0.12‒0.82	［^97^］
Ag-dcp	TCH （fish）	15	0.14	96.3‒100.9	0.6‒1.8	［^98^］
EuMOF-FITC	histamine （fish）	10	19.52	\	\	［^99^］
Eu-BOP/BDC-OH	histamine （shrimp）	20	1.53	98.71‒103.58	0.70‒4.66	［^100^］
Zn/Eu-MOF	ofloxacin （fish）	0.5	0.44	100.2‒112.1	1.17‒1.55	［^101^］
Fe/Eu–MOF	diethylstilbestrol （shrimp）	immediate	0.00663	92.14‒97.93	0.69‒2.84	［^102^］
CDs@Fe/Zr-MOF	BA （shrimp）	0.5	0.17，2.95	\	\	［^103^］

CDs： carbon dots； GSH： glutathione； NCs： nanoclusters； BTEC： benzene-1，2，4，5-tetracarboxylic acid； RhB： rhodamine B； DDB： 1，3-di（3′，5′-dicarboxylphenyl）benzene； dcp： 3-（3，5-dicarboxyphenyl）-6-carboxypyridine； FITC： fluorescein isothiocyanate； BOP： boronoisophthalic acid； TCH： tetracycline hydrochloride.

### 2.3 表面增强拉曼散射技术

表面增强拉曼散射（SERS）是一种无标记的检测方法，利用金属纳米结构通过强等离子体共振（LSPR）效应产生的拉曼增强特性提供独特的分子指纹信息。传统SERS基底存在易团聚和活性不理想的局限^［104］^，而MOFs/贵金属纳米颗粒的复合材料基底既具有MOFs的预浓缩效应，还能通过结构柔性增强SERS基底的电荷转移作用，增强SERS信号^［105］^。

Au@MIL-100（Fe）核壳结构利用MIL-100（Fe）壳层的高吸附能力富集目标分析物，并通过Au核的局域LSPR特性增强拉曼信号，其中MIL-100（Fe）作为稳定剂可保护金纳米粒子不发生聚集。将该混合SERS基底滴加至活鱼表面，可以实现对鱼体表面孔雀石绿的原位检测^［106］^。在Au@MIL-100（Fe）的基础上以磁性纳米颗粒作为核心，使材料的分布更加均匀，形成具有核壳卫星结构的强稳定性SERS基底^［107］^。

大部分SERS传感器主要利用拉曼光谱指纹区的特征峰信号，但易产生光谱重叠干扰的问题，而低波数拉曼静默区（10~500 cm^-1^）应用较少。Chen等^［108］^开发了基于金纳米双锥（Au NBPs）封装ZIF-8的新型低波数SERS传感器（图5），H_2_S可通过竞争吸附导致Au-Br键信号变化，实现低波数的拉曼信号变化。该传感器无需预处理即可检测腐败鱼肉释放的H_2_S，且能够在5 min内完成富集与信号采集。

**图5:**
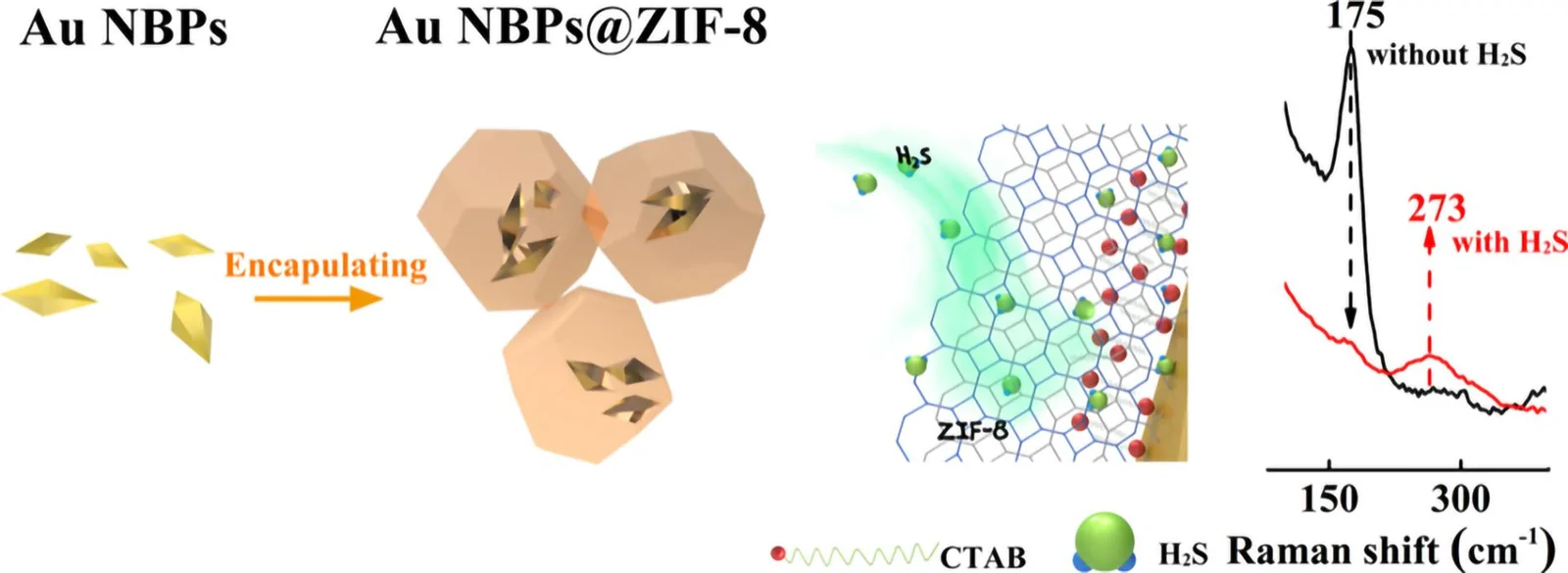
基于Au NBPs@ZIF-8的低波数SERS传感器用于H_2_S气体检测的机理^［108］^

综上，基于MOFs材料的表面增强拉曼散射技术在水产品危害物分析中的应用归纳如表6所示。

**表6:** 基于MOFs材料的表面增强拉曼散射技术在水产品危害物分析中的应用

Main material	Analyte （samples）	Analytical time/min	LOD/（nmol/L）	Recovery/%	RSD/%	Ref.
Au@MIL-100（Fe）	MG （fish）	<1	8.0	\	\	［^106^］
MNP@Au@MIL-100（Fe）	MG （shrimp）	5	0.132	\	\	［^107^］
Au@ZIF-8	bisphenol A （fish）	3	438	99.68‒102.35	3.28‒7.58	［^109^］
Au NBPs@ZIF-8	H_2_S （fish）	5	0.17	\	\	［^108^］
Ag@ZIF-8@Au	diazepam （fish，crab，lobster）	0.1	0.443	85.3‒111.9	2.39‒9.35	［^110^］
Au NFs@ZIF-67	histamine （fish）	<1	87	93.42‒102.23	2.21‒4.83	［^111^］
Fe_3_O_4_@ZIF-67@Ag	MG （tilapia）	10	0.516	80.0‒106.0	0.55‒4.69	［^112^］

MNP： magnetic nanoparticle； NBPs： nanobipyramids； NFs： nanoflowers.

### 2.4 免疫学识别技术

免疫学识别技术基于抗原-抗体识别，常与电化学和荧光等检测原理联用，具有特异性高和操作简便等优势。MOFs的多孔结构、超高比表面积和多功能位点使其可以有效地与抗体结合，而良好的生物相容性还可以有效固定生物分子，因此已被广泛用于免疫分析^［113］^。

#### 2.4.1 电化学免疫识别技术

ZIF-8/AuNPs复合材料可用作固定抗体和抗原的平台。其中金纳米粒子提供生物相容性和优异的电子传导性，ZIF-8则在提供吸附性能的同时作为优良的稳定剂，能够将金纳米粒子牢固地锚定在其结构中。采用硫堇和二茂铁分别标记嗜水气单胞菌和铜绿假单胞菌的抗体，并固定于ZIF-8/AuNPs平台，能够实现受污染的鱼组织中2种食源性病原体的电化学免疫同时检测（图6）^［114］^。免疫方法多采用来自哺乳动物的IgG抗体，而卵黄抗体（IgY）具有与IgG相当甚至更高的灵敏度，同时具有成本低且便于大规模生产的特性。将二茂铁/苯硼酸-UiO-66-N_3_复合探针与卵黄抗体结合共同识别捕获金黄色葡萄球菌，并通过二茂铁的电化学氧化还原信号进行检测，在虾肉样品中实现了99.5%~110.0%的回收率^［115］^。

**图6:**
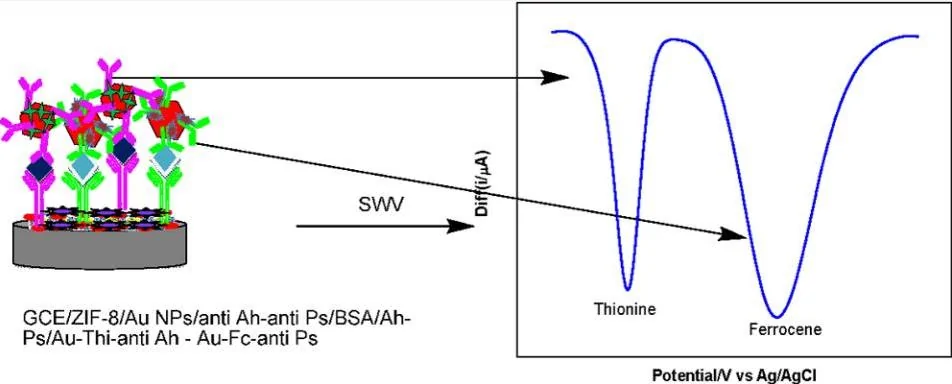
通过GCE/ZIF-8/AuNPs对嗜水气单胞菌和铜绿假单胞菌进行电化学检测的过程^［114］^

#### 2.4.2 荧光免疫识别技术

镧系元素具有荧光寿命长、荧光强度高、信噪比高和光漂白率低等特性。将镧系金属Eu^3+^通过后合成修饰引入到UiO-66（COOH）_2_中，能够同时结合MOFs的固有优势和镧系元素的荧光特性，MOFs刚性骨架结构还能保护抗体活性。将其用于虾样品中五氯苯酚钠的免疫层析检测，检测时间仅为2 min，灵敏度相较已报道的方法提升了10倍^［116］^。利用定向抗体修饰并负载荧光量子点的ZIF-8复合材料开发便携式荧光生物传感器，用于检测海鲜中的河豚毒素，在河豚和蛤蜊样品中实现92.37%~108.94%的回收率^［117］^。

#### 2.4.3 双模式免疫识别技术

单模式分析得到的结果易受环境等多种因素影响，而双模式分析输出的两种独立响应信号可以相互验证，提高结果准确性^［113］^。

锆基有机框架PCN-222具有高稳定性、良好的生物相容性以及易于合成等优点，在紫外光激发下能够发射出强烈的红色荧光；该材料本身具有颜色，可同时用于比色检测。PCN-222与抗体结合可构建双信号的免疫层析法，用于快速灵敏测定虾样品中呋喃唑酮代谢物的衍生物CPAOZ，灵敏度较胶体金法提高5~10倍^［118］^。通过修饰和改性可进一步提高材料性能，例如脂质体的修饰能有效提高PCN-222的生物相容性和水溶性，增强其荧光强度和寿命^［119］^；果胶改性则可以改善PCN-222的疏水性，并降低其潜在卟啉泄漏风险；基于果胶改性PCN-222的双模式侧流免疫层析法（LFIA）已成功应用于虾样品中呋喃西林代谢物氨基脲的衍生物CPSEM的检测^［120］^。

综上，基于MOFs材料的免疫学识别技术在水产品危害物分析中的应用归纳如表7所示。

**表7:** 基于MOFs材料的免疫学识别技术在水产品危害物分析中的应用

Main material	Analytical method	Analyte （samples）	Analytical time/min	LOD/（ng/mL）	Recovery/%	Ref.
UiO-66-Eu	fluorescence	PCP （shrimp）	2	0.37	79.7‒108.3	［^116^］
UiO-66-N_3_@Fc-PBA	electrochemistry	*S. aureus* （shrimp）	20	\	99.5‒110.0	［^115^］
ZIF-8/AuNPs	electrochemistry	pathogen （fish）	45	\	88.75‒106.97	［^114^］
PCN-222-mAbs	colorimetric，fluorescence	CPAOZ （shrimp）	12	0.5‒1.0	92.34‒117.44	［^118^］
PCN-222@liposome	colorimetric，fluorescence	sulfonamide （shrimp）	12	0.01‒0.1	92.09‒118.64	［^119^］
PCN-222@Pectin	colorimetric，fluorescence	CPSEM （shrimp）	10‒15	0.05‒0.1	92.6‒119.5	［^120^］
NU-902@HRP	colorimetric	CAP （shrimp）	38	0.0184	84.71‒107.65	［^121^］
QD@Zn-MOF*Ab	fluorescence	TTX （puffer fish，clam）	15	0.13‒0.4	92.37‒108.94	［^117^］

Fc： ferrocene； PBA： phenylboronic acid； mAbs： monoclonal antibodies； HRP： horseradish peroxidase； PCP： sodium pentachlorophenate； *S. aureus*： *Staphylococcus aureus*； CPAOZ： 3-｛［（4-carboxyphenyl）methylene］amino｝-2-oxazolidinone； CPSEM： 4［（4-carboxyphenyl）-methylene］-hydrazinecarboxamide； TTX： tetrodotoxin.

### 2.5 适配体识别技术

作为人工合成的识别分子，适配体（Apt）不仅可精准识别目标物的分子结构，还能针对抗体难以识别的小分子目标物进行适配体的筛选，相比抗体成本更低且结果更加稳定^［122］^，且同样可结合电化学和荧光等多种检测原理。MOFs的多孔结构可促进待测物高效到达识别位点，提高相互作用的效率；孔径经调节后可以排除干扰物质，增强对目标分析物的选择性^［123］^。

#### 2.5.1 电化学适配体识别技术

Cu-BTC具有高比表面积和良好的机械稳定性，为适配体固定提供了理想平台；其中Cu^2+^具有优异的电化学活性，可在电极界面提供稳定的电化学信号。掺杂高导电性的聚苯胺可以增强Cu-BTC的电化学响应，通过配位作用将适配体组装到电极表面，可实现草鱼样品中四环素的适配体传感检测^［124］^。UiO-66-NH_2_表面富含氨基活性位点，通过戊二醛交联剂共价固定适配体的互补DNA，增强生物分子的稳定性；结合多壁碳纳米管和还原氧化石墨烯纳米带（MWCNT@rGONR）的高导电性，能够解决现有适配体传感器稳定性差和电信号弱的缺陷，实现鱼肉中卡那霉素的电化学生物传感检测^［125］^。

除了直接使用，MOFs还可以作为优良的模板制备具有中空结构的吸附材料^［126］^。以具有高导电性和稳定性的棒状稀土MOFs（Gd_4_-MOF）为基础，通过牺牲模板法生成具有更优响应电流的普鲁士蓝；汞与DNA适配体特异性结合形成T-Hg-T结构，导致构象变化并引起响应电流改变，由此实现水产品中痕量汞的检测^［127］^。

#### 2.5.2 荧光适配体识别技术

传统多色发光材料毒性高且易光漂白，而2D MOFs能高效猝灭荧光染料标记的单链DNA，显著降低背景荧光并提高信噪比，为生物分子探针提供了新思路。当单链DNA与互补链形成双链DNA时，荧光可几乎完全恢复。将2D-MOF（Cu-TCPP）荧光传感系统与双搅拌棒辅助靶标替换系统结合，能够实现无酶信号放大；应用于鱼类组织切片中氯霉素、土霉素和卡那霉素的检测时，可明显观察到抗生素的荧光成像结果^［128］^。PCN-222可强吸附适配体，当适配体被荧光染料标记时，PCN-222即可实现高效荧光猝灭；若目标物存在，适配体从PCN-222表面释放，导致荧光恢复。该方法成功应用于虾样品中氯霉素检测，检出限为0.08 pg/mL^［129］^。

将邻苯二胺（OPD）作为底物，ZrFe-MOF的类过氧化物酶活性可催化OPD氧化生成荧光产物OPDox，同时猝灭自身固有荧光。适配体通过Zr中心的磷酸特异性位点吸附于材料表面后，会抑制其类过氧化物酶活性，导致固有荧光恢复、OPDox的荧光减弱；当存在目标物时，适配体与目标物特异性结合，重新激活ZrFe-MOF的类过氧化物酶活性，使两个荧光信号呈相反变化。构建的比率型荧光适配体传感器成功应用于河豚和蛤蜊样品中河豚毒素的测定，检出限为0.027 ng/mL^［130］^，优于国标方法检出限1 ng/mL。

#### 2.5.3 多模式适配体识别技术

AuNPs@MIL-101结合MIL-101的选择性吸附能力，以及AuNPs的SERS活性增强能力，可用于构建荧光-SERS双模式适配体传感器。针对河豚毒素的检测，传感器以标记荧光和拉曼信号基团的河豚毒素适配体为探针，利用MIL-101的选择性吸附能力将适配体吸附于AuNPs@MIL-101材料表面，同时产生荧光猝灭和SERS增强效应；当河豚毒素与适配体结合后，适配体被释放，导致荧光信号恢复、SERS信号减弱^［131］^。

Zhan等^［132］^通过计算模拟，设计出亲和力提升且合成成本降低的截短适配体，结合IRMOF-3@PtNPs-TB复合物构建荧光/比色/SERS三模式适配体传感器。IRMOF-3具有来源于NH_2_-BDC配体的本征荧光，而PtNPs用于催化显色反应完成比色检测，再结合甲苯胺蓝（TB）的拉曼响应实现SERS检测。该三模式传感器在检测水产样品里的氧氟沙星时，检出限为0.040 3~0.345 0 ng/mL。

综上，基于MOFs材料的适配体识别技术在水产品危害物分析中的应用总结见表8。

**表8:** 基于MOFs材料的适配体识别技术在水产品危害物分析中的应用

Main material	Analytical method	Analytes （samples）	Analytical time/min	LOD/（pmol/L）	Recovery/%	Ref.
IRMOF-3@PtNPs-TB	colorimetric，fluorescence，SERS	ofloxacin （fish，shrimp）	130	111.5‒954.7	89.2‒103.8	［^132^］
MOF-76-Apt@MIP	fluorescence	MG （fish）	15‒30	303.5	95.0‒105.6	［^133^］
MIL-53（Al）@CdTe	electrochemistry	Hg（Ⅱ），Pb（Ⅱ） （fish，shrimp）	60	4.1，24‒37	91‒103	［^134^］
AuNPs@MIL-101	fluorescence，SERS	TTX （puffer fish，clam）	42	18.79‒25.06	97.17‒105.7	［^131^］
Fe_3_O_4_@MIL-101@AuNPs	fluorescence	okadaic acid （shellfish）	20	0.0186	88.2‒107.5	［^135^］
Fe_3_O_4_@UiO-66-apt，Au@Fc-PBA	electrochemistry	V.P （shrimp）	20	\	94.0‒107	［^136^］
UiO-66-NH_2_/MCA/MWCNT@rGONR	electrochemistry	kanamycin （fish）	35	13000	98.3‒107.7	［^125^］
PCN-222	fluorescence	CAP （shrimp）	26	0.25	89.91‒106.15	［^129^］
Cu-TCPP	fluorescence	antibiotic （fish）	30	0.9‒2.4	97.9‒110	［^128^］
PAN@Cu-BTC	electrochemistry	TC （fish）	60	0.32	90.0‒102.0	［^124^］
TC-Apt/Ce-MOF-808@CeO_2_/CS/SpE	electrochemistry	TC （shrimp）	35	0.21	96.2‒104	［^137^］
Zr-MOF-Apt	fluorescence	biotoxin （shellfish）	20	1170‒3070	95.97‒103.22	［^138^］
ZrFe-MOF	fluorescence	TTX （puffer fish，clam）	50	85	93.74‒107.53	［^130^］
ZrO_2_@Ni/Co-MOFs@AuNPs	electrochemistry	Cd（Ⅱ） （scallop）	30	0.00170	89.01‒108.63	［^139^］
Fe_3_O_4_@MOF（Fe-Cu）-GNS-MBA-Apt	SERS	V.P （shrimp）	55	\	89.3‒96.7	［^140^］

TB： toluidine blue； MCA： melamine and cyanuric acid monomers； rGONR： reduced graphene oxide nanoribbon； TC： tetracycline； CeO_2_： cerium dioxide； SpE： screen-printed electrode； GNS： graphene nanosheets； MBA： mercaptobenzoic acid； Gd_4_： tetranuclear gadolinium cluster； V.P： *Vibrio parahaemolyticus*.

### 2.6 分离-检测一体化技术

MOFs的多功能性使其成为一体化策略的理想材料。通过使用MOFs等多功能材料，以及利用微流控芯片^［141］^或集成传感器等一体化平台，可以将样品前处理与检测这两个传统上分离的步骤集成到同一流程或设备中。例如，MSPE常与SERS结合^［142，143］^，在消除干扰的同时，还能通过磁性复合材料增强SERS信号。分离-检测一体化的设计可以减少样品转移等步骤，简化流程，提升分析速度，还可以避免人工操作带来的误差，提高结果的准确性；同时，促进了便携式检测设备的开发。

MIL-100（Fe）具有良好的化学稳定性，其配体可实现生物降解，无二次污染。将MIL-100（Fe）以聚偏氟乙烯（PVDF）为黏合剂固定于三聚氰胺海绵（MeS），可以解决MIL-100（Fe）粉末易团聚、难回收的缺陷。通过MeS/PVDF/MOF复合材料可将固相萃取技术与紫外检测相结合，构建吸附-检测一体化平台，实现鱼样中孔雀石绿的高效检测，检出限达2.29 μg/L^［144］^。

在柔性碳纸（CP）上生长镍基导电MOFs（CMOF），并通过可控电沉积结合金纳米颗粒，能够形成基于CP-CMOF@Au芯片的SERS一体化平台，用于检测水产品中的地西泮^［145］^。其中的CMOF通过预浓缩效应提高了方法灵敏度，同时通过导电性解决了传统MOFs基底分布不均匀的问题。碳纸价格低廉，可同时作为基底材料和电沉积的工作电极；其柔性能适应多种样品收集方法，从而提高了SERS平台的适用性。

## 3 结论

MOFs在水产品危害物的快速检测领域中显示出巨大潜力，但从实验室研究到实际应用时仍面临一系列挑战。首先是MOFs材料的设计与性能优化。在稳定性方面，部分MOFs由于水稳定性和化学稳定性不足，在复杂生物基质中易发生骨架水解或配体脱落，导致结构坍塌和性能下降。构建高稳定性MOFs或将MOFs与碳材料、聚合物等稳定材料复合是当前重要的解决途径。在选择性方面，多数MOFs的孔径仅有微孔级别，对于大分子毒素或致病菌等危害物的捕获能力有限；同时，复杂基质中结构相似物的共存也对MOFs的分子识别精度提出了更高要求。通过孔径工程构建介孔或多级孔MOFs，以及通过分子印迹聚合物或适配体的功能化引入特异性识别位点是提升选择性的有效策略。其次，多物质的同时分析仍面临挑战。水产品中危害物种类多样，物理化学性质差异显著，难以通过单一MOFs材料实现对多种目标物的高效富集。多物质共吸附可能导致信号相互干扰，影响检测准确性。发展混合MOFs材料或结合阵列传感器，是应对复杂样品多残留分析的重要方向。最后，材料的成本与规模化生产问题不容忽视。多数MOFs的合成依赖昂贵的有机配体和有机溶剂，且要求高温高压条件，难以满足商业化需求。不同批次合成的MOFs在结晶度和粒径等方面存在差异，导致结果的重现性难以保证。开发水相合成和室温合成等绿色温和合成方法，并建立标准化质量控制体系，是推动MOFs走向实际应用的关键。未来，基于MOFs的水产品危害物分离分析技术将继续朝着分离-检测一体化的方向发展。持续深化MOFs及其复合材料的相关研究，并据此开发一体化技术，有望为构建高效便捷的水产品安全监管体系提供有效的解决方案。
